# M-COPA suppresses endolysosomal Kit-Akt oncogenic signalling through inhibiting the secretory pathway in neoplastic mast cells

**DOI:** 10.1371/journal.pone.0175514

**Published:** 2017-04-12

**Authors:** Yasushi Hara, Yuuki Obata, Keita Horikawa, Yasutaka Tasaki, Kyohei Suzuki, Takatsugu Murata, Isamu Shiina, Ryo Abe

**Affiliations:** 1 Division of Immunobiology, Research Institute for Biomedical Sciences, Tokyo University of Science, Noda, Chiba, Japan; 2 Department of Applied Chemistry, Faculty of Science, Tokyo University of Science, Shinjuku-ku, Tokyo, Japan; Emory University, UNITED STATES

## Abstract

Gain-of-function mutations in Kit receptor tyrosine kinase result in the development of a variety of cancers, such as mast cell tumours, gastrointestinal stromal tumours (GISTs), acute myeloid leukemia, and melanomas. The drug imatinib, a selective inhibitor of Kit, is used for treatment of mutant Kit-positive cancers. However, mutations in the Kit kinase domain, which are frequently found in neoplastic mast cells, confer an imatinib resistance, and cancers expressing the mutants can proliferate in the presence of imatinib. Recently, we showed that in neoplastic mast cells that endogenously express an imatinib-resistant Kit mutant, Kit causes oncogenic activation of the phosphatidylinositol 3-kinase-Akt (PI3K-Akt) pathway and the signal transducer and activator of transcription 5 (STAT5) but only on endolysosomes and on the endoplasmic reticulum (ER), respectively. Here, we show a strategy for inhibition of the Kit-PI3K-Akt pathway in neoplastic mast cells by M-COPA (2-methylcoprophilinamide), an inhibitor of this secretory pathway. In M-COPA-treated cells, Kit localization in the ER is significantly increased, whereas endolysosomal Kit disappears, indicating that M-COPA blocks the biosynthetic transport of Kit from the ER. The drug greatly inhibits oncogenic Akt activation without affecting the association of Kit with PI3K, indicating that ER-localized Kit-PI3K complex is unable to activate Akt. Importantly, M-COPA but not imatinib suppresses neoplastic mast cell proliferation through inhibiting anti-apoptotic Akt activation. Results of our M-COPA treatment assay show that Kit can activate Erk not only on the ER but also on other compartments. Furthermore, Tyr568/570, Tyr703, Tyr721, and Tyr936 in Kit are phosphorylated on the ER, indicating that these five tyrosine residues are all phosphorylated before mutant Kit reaches the plasma membrane (PM). Our study provides evidence that Kit is tyrosine-phosphorylated soon after synthesis on the ER but is unable to activate Akt and also demonstrates that M-COPA is efficacious for growth suppression of neoplastic mast cells.

## Introduction

Kit, a cell-surface receptor for stem cell factor (SCF), belongs to the type III receptor tyrosine kinase family that includes platelet-derived growth factor receptor α/β (PDGFRα/β), Flt3, and Fms [1–4]. It plays a pivotal role in the development of mast cells, interstitial cells of Cajal (ICC), hematopoietic cells, germ cells, and melanocytes [3–5].

Kit is composed of five N-glycosylated immunoglobulin domains in the N-terminal extracellular portion that bind SCF, a transmembrane domain, and the carboxy-terminal intracellular tyrosine kinase domain [4–6]. The binding of SCF autophosphorylates Kit on specific tyrosine residues (Tyr), such as Tyr568/570, Tyr703, Tyr721, and Tyr936 [5–8]. Kit then binds to other cytoplasmic enzymes, such as PI3K and Src kinases, and this complex activates downstream molecules [5–9]. This activates the Akt-Bad pathway and the Ras-Mek-Erk cascade, which regulate gene expression and cytoskeletal structures, resulting in cell proliferation and survival [7–11].

In many mast cell neoplasms and GISTs, Kit develops gain-of-function mutations, causing permanent, ligand-independent activation of the receptor [12–15]. Mutant Kit transforms mast cells and ICC through permanent activation of the PI3K-Akt pathway and STATs resulting in the development of mast cell tumours and GISTs [16–20]. Thus, the drug imatinib, a selective inhibitor of Kit, improved the prognosis of GIST patients [15,21,22]. However, imatinib treatment is ineffective in most cases of mast cell tumours because they express imatinib-resistant Kit that has a mutation in the kinase domain [12,13,23,24]. Furthermore, GISTs frequently acquire a secondary mutation in the Kit kinase domain, resulting in imatinib resistance [15,25]. Considering that other cancer-causing receptors develop drug resistance in a manner similar to imatinib-resistant Kit [26], a new strategy for inhibition of receptors bearing a mutation in the kinase domain is desirable.

**Fig 1:**
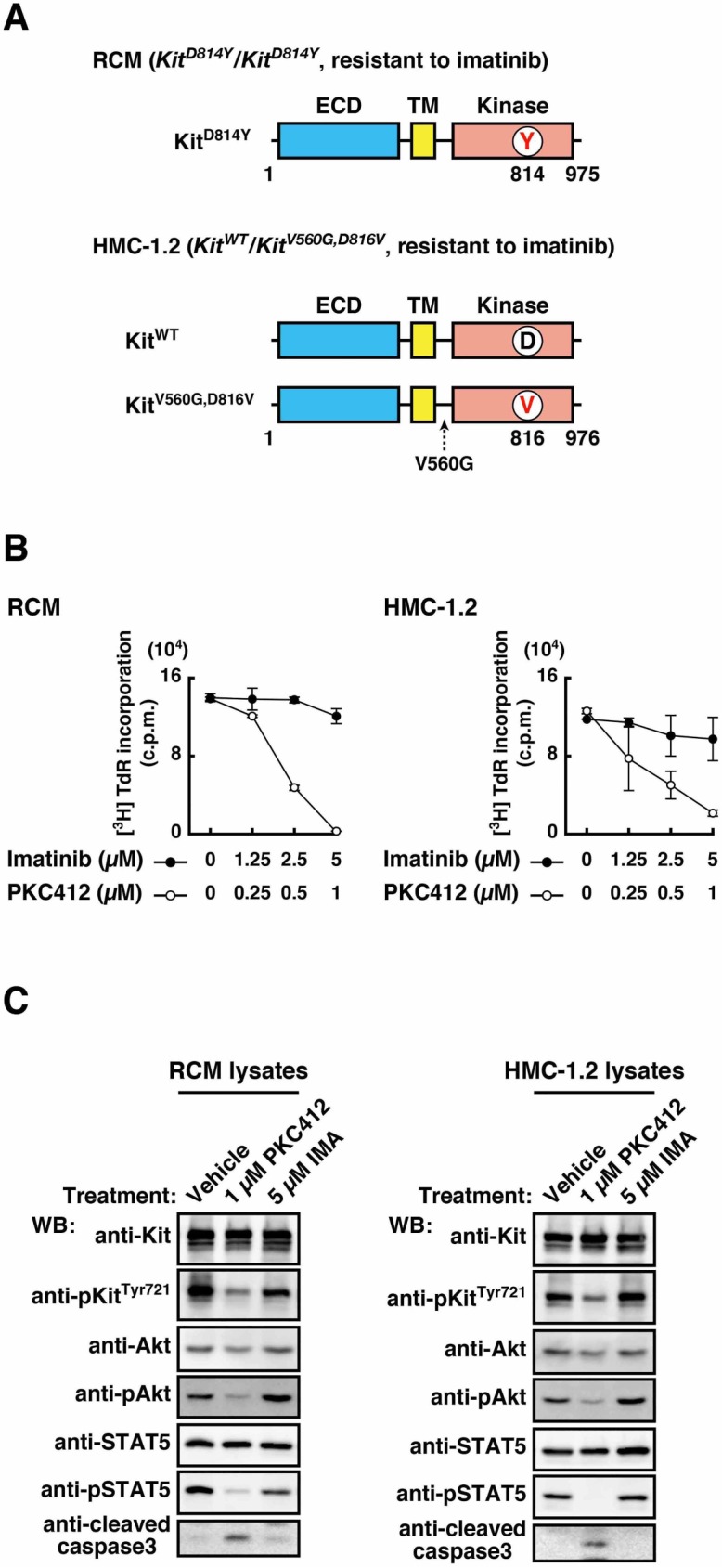
Mutations in the Kit kinase domain confer imatinib resistance to neoplastic mast cells. (**A**) Schematic representations of wild-type Kit (Kit^WT^) and mutant Kit showing the extracellular domain (ECD), the transmembrane domain (TM), and the kinase domain. Asp814 and Asp816, D in black; Tyr814 and Val816, Y and V in red. In addition to D816V, V560G is found in HMC-1.2’s Kit, but we did not find a difference between RCM and HMC-1.2 in Kit localization and oncogenic signalling. (**B**) RCM (left) and HMC-1.2 (right) were treated with vehicle (0), imatinib (Kit kinase inhibitor, closed circles), or PKC412 (inhibits type III tyrosine kinase, open circles) for 24 hours. Proliferation was assessed by [^3^H]-thymidine incorporation. Results (c.p.m.) are means ± SD (*n* = 3). (**C**) RCM and HMC-1.2 were treated with vehicle, 1 μM PKC412 or 5 μM imatinib (IMA) for 16 hours. Cell lysates were immunoblotted with anti-Kit, anti-phospho-Kit^Tyr721^ (anti-pKit^Tyr721^), anti-Akt, anti-pAkt, anti-STAT5, anti-pSTAT5, and anti-cleaved caspase-3. Note that imatinib did not affect the phosphorylation of Kit or cell proliferation.

In human neoplastic mast cell disorders such as mastocytosis and mast cell leukemia, Kit often has an Asp816Val substitution in the kinase domain (D816V) [12,13,27] (see Fig 1A). Similar mutations are also found in mouse mastocytoma (eg, D814Y and D814V) [12,13,28]. We recently reported that Kit^D814Y^ activates the PI3K-Akt pathway and STAT5 on endolysosomes and the ER, respectively [29]. Furthermore, a recent study described a new inhibition strategy for Flt3 receptor kinase bearing a mutation in the kinase domain through blocking the receptor trafficking with fluvastatin [30]. These findings provided evidence that intracellular trafficking of mutant receptors is a promising target for the treatment of cancers.

M-COPA is a novel inhibitor of ADP-ribosylation factor 1 (ARF1), which plays a role in vesicular trafficking [31,32]. Recent studies showed that M-COPA blocks the trafficking of Met receptor kinase along the secretory pathway to the PM, resulting in suppression of receptor-ligand binding [33,34]. Thus, M-COPA has an anti-tumour effect *in vivo*. However, whether M-COPA inhibits oncogenic signalling of mutant receptors that are localized on organelles such as endolysosomes is unknown.

Here, we show that in neoplastic mast cells from mice and humans, M-COPA significantly inhibits the biosynthetic trafficking of imatinib-resistant Kit mutant (hereafter, referred to as Kit^mut^) from the ER. In M-COPA-treated cells, Kit^mut^ is unable to activate Akt because endolysosomal Kit^mut^ disappears. Since Kit^mut^ activates STAT5 selectively on the ER, M-COPA enhances STAT5 activation. Importantly, M-COPA but not imatinib suppresses neoplastic mast cell proliferation through inhibiting the anti-apoptotic Akt activation that occurs only on endolysosomes. Results of our M-COPA treatment assay show that Kit^mut^ can activate Erk not only on the ER but also on other organelles. Furthermore, Kit Tyr568/570, Tyr703, Tyr721, and Tyr936 are phosphorylated on the ER, indicating that these five tyrosine residues are all phosphorylated on the ER before Kit^mut^ reaches the PM. Our study provides evidence that Kit^mut^ is tyrosine-phosphorylated soon after synthesis on the ER but is unable to activate Akt and also demonstrates that M-COPA is efficacious for growth suppression of neoplastic mast cells.

## Materials and methods

### Cell culture

RCM (R cell mutant Kit) cells [29] were established from splenocytes of DO11.10 mice by repeated stimulation with ovalbumin peptides *in vitro*. This cell line exhibits a mast cell-like surface phenotype, Kit^+^ FcεRI^+^, and mast cell-like expression profiles of proteases. Moreover, RCM cells can secrete biologically active products, such as histamine and β-hexosaminidase, upon stimulation. They homozygously express Kit^D814Y^ and proliferate autonomously. HMC-1.2 (human mast cell line-1.2) cells [27] were established from a patient with mast cell leukemia. The cell line heterozygously expresses Kit^V560G,D816V^ and shows autonomous proliferation. Both cell lines were cultured at 37°C in RPMI1640 medium supplemented with 10% fetal calf serum (FCS), penicillin, streptomycin, glutamine (Pen/Strep/Gln), and 50 μM 2-mercaptoethanol. A lung adenocarcinoma cell line A549 was purchased from American Type Culture Collection (Manassas, VA, USA), and the cells were cultured at 37°C in RPMI1640 medium supplemented with 10% FCS, and Pen/Strep/Gln.

### Cell proliferation assay

RCM and HMC-1.2 cells were cultured with inhibitors for 16 hours, and then treated with [^3^H]-thymidine deoxyribonucleotide (TdR) for 8 hours. Cell proliferation was evaluated by the incorporation of [^3^H]-TdR.

### Antibodies

The following antibodies were purchased: Kit (M-14), STAT5 (C-17), Erk2 (K-23) and cathepsin D (H-75) from Santa Cruz Biotechnology (Dallas, TX); Kit[pTyr719], Kit[pTyr703], Akt (40D4), Akt[pT308] (C31E5E), STAT5[pTyr694] (D47E7), Erk[pThr202/pTyr204] (E10), and cleaved caspase-3 from Cell Signaling Technology (Danvers, MA); p85, phospho-tyrosine (4G10), and Kit[pTyr568/570] from Millipore (Billerica, MA); LAMP1 from Sigma (St. Louis, MO); Kit[pTyr936] from Thermo Scientific Pierce (Rockford, IL) and calnexin from Enzo Life Sciences (Farmingdale, NY). HRP-labeled anti-mouse Ig, anti-rabbit Ig, and anti-goat Ig secondary antibodies were purchased from the Jackson Laboratory (Bar Harbor, MA). Alexa-Fluor-conjugated secondary antibodies were obtained from Molecular Probes (Eugene, OR).

### Chemicals

Imatinib (Cayman Chemical, Ann Arbor, MI), PKC412 (Santa Cruz Biotechnology), and Akt inhibitor VIII (Millipore) were dissolved in dimethylsulfoxide (DMSO). Monensin (Biomol, Exeter, UK), brefeldin A (Wako, Osaka, Japan), and bafilomycin A1 (Sigma) were dissolved in ethanol. M-COPA was synthesized as previously described [31] and dissolved in DMSO.

### Immunofluorescence confocal microscopy

Cells were fixed with methanol for 10 minutes at -20°C, then cyto-centrifuged onto coverslips. Fixed cells were permeabilized and blocked for 30 minutes in PBS supplemented with 0.1% saponin and 3% BSA, and then incubated with a primary and secondary antibody for 1 hour each. After washing with PBS, cells were mounted with Fluoromount (DiagnosticBioSystems, Pleasanton, CA). Confocal images were obtained with a Fluoview FV10i laser scanning microscope with an x60 1.20 N.A. water-immersion objective (Olympus, Tokyo, Japan). Composite figures were prepared with Photoshop Elements 10 and Illustrator CS6 software (Adobe, San Jose, CA). Pearson’s R correlation coefficients were calculated with NIH ImageJ 1.48v software.

### Immunoprecipitation and western blotting

Lysates from 1~2 x 10^6^ cells were prepared in SDS-PAGE sample buffer or NP-40 lysis buffer (50 mM HEPES, pH 7.4, 10% glycerol, 1% NP-40, 4 mM EDTA, 100 mM NaF, 1 μg/ml aprotinin, 1 μg/ml leupeptin, 1 μg/ml pepstatin A, 1 mM PMSF, and 1 mM Na_3_VO_4_). Immunoprecipitation was performed at 4°C for 5 hours using protein G pre-coated with antibody. Immunoprecipitates were dissolved in SDS-PAGE sample buffer, subjected to SDS-PAGE, and electro-transferred onto PVDF membranes. Immunodetection was performed by ECL (PerkinElmer, Waltham, MA). Sequential re-probing of membranes was performed after the complete removal of primary and secondary antibodies in stripping buffer, or by inactivation with peroxidase in 0.1% NaN_3_. Results were analyzed with an LAS-3000 image analyzer with Science Lab software (Fujifilm, Tokyo, Japan) or with a c-Digit imaging system with Image Studio Digit software (Licor Biosciences, Lincoln, NE).

### Analysis of protein glycosylation

Following the manufacturer’s instructions (New England Biolabs, Ipswich, MA), NP-40 cell lysates were treated with endoglycosidase H (endo H) or peptide-N-glycosidase F (PNGase F) for 1 hour at 37°C. The reactions were stopped with SDS-PAGE sample buffer, and products were resolved by SDS-PAGE and immunoblotted.

### Gene silencing of *Kit*^*D814Y*^ with small interfering RNAs (siRNAs) and electroporation

For silencing *Kit*^*D814Y*^, siRNA duplexes were purchased from Sigma (Kit1: GAAGGAUUAUGUCAAAUCUTT, Kit2: GACAUGAAGCCUGGCGUUUTT). The control siRNA duplex was also purchased from Sigma (Mission negative control SIC-001). For knockdown of *Kit*^*D814Y*^, cells were transfected using a Gene Pulser II electroporation system (Bio-Rad Laboratories, Hercules, CA) and cultured for 20 hours.

### Statistical analyses

For statistical analysis, experiments were repeated as three biological replicates. Differences between two or more groups were analyzed by one-way analysis of variance (ANOVA) followed by Dunnett’s multiple comparison *post-hoc* test. All significant differences showed a 5% level of probability.

## Results

### RCM and HMC-1.2 can proliferate in the presence of imatinib due to mutations in the Kit kinase domain

We recently established a mast cell line from mouse splenocytes, made up of RCM cells bearing Kit and FcεRI [29]. These cells grow without cytokines and develop tumours *in vivo*. RCM cells homozygously express Kit^D814Y^, an imatinib-resistant mutant [29] (Fig 1A). First, to confirm whether the proliferation of RCM was imatinib-resistant, we performed an [^3^H]-thymidine deoxyribonucleotide ([^3^H]-TdR) uptake assay. Fig 1B shows that RCM cells proliferated in the presence of imatinib at any of the concentrations used (closed circles). Similarly, the proliferation of HMC-1.2 (a human mast cell line established from a mast cell leukemia patient), which heterozygously express Kit^V560G,D816V^ [12,13,27] (Fig 1A), was unaffected by imatinib treatment (Fig 1B, right, closed circles). These results indicate that imatinib does not have an inhibitory effect on growth of these neoplastic mast cells. In support of this, Western blotting analysis showed that the phosphorylation of Kit as well as of Akt and STAT5 was unaffected by imatinib treatment (Fig 1C). As previously reported [16,18,29,35], Kit^mut^ knockdown by siRNAs and Kit inhibition by PKC412 (multi-tyrosine kinase inhibitor) greatly inhibited the growth of RCM and HMC-1.2 (Fig 1B, open circles and S1 Fig), confirming Kit-dependent growth of these cells. In addition, PKC412 markedly decreased the activation of Kit^mut^, resulting in inhibition of Akt and STAT5 (Fig 1C). Inhibition of Kit^mut^ by PKC412 can induce cleavage of caspase-3, a sign of apoptosis [16,19], but imatinib did not have the same effect (Fig 1C, lower panels). Taken together, the results suggest that these cells proliferate in the presence of imatinib in a manner dependent on their Kit kinase domain mutant.

### M-COPA, a novel inhibitor of the secretory pathway, blocks biosynthetic transport of Kit^mut^ from the ER

**Fig 2:**
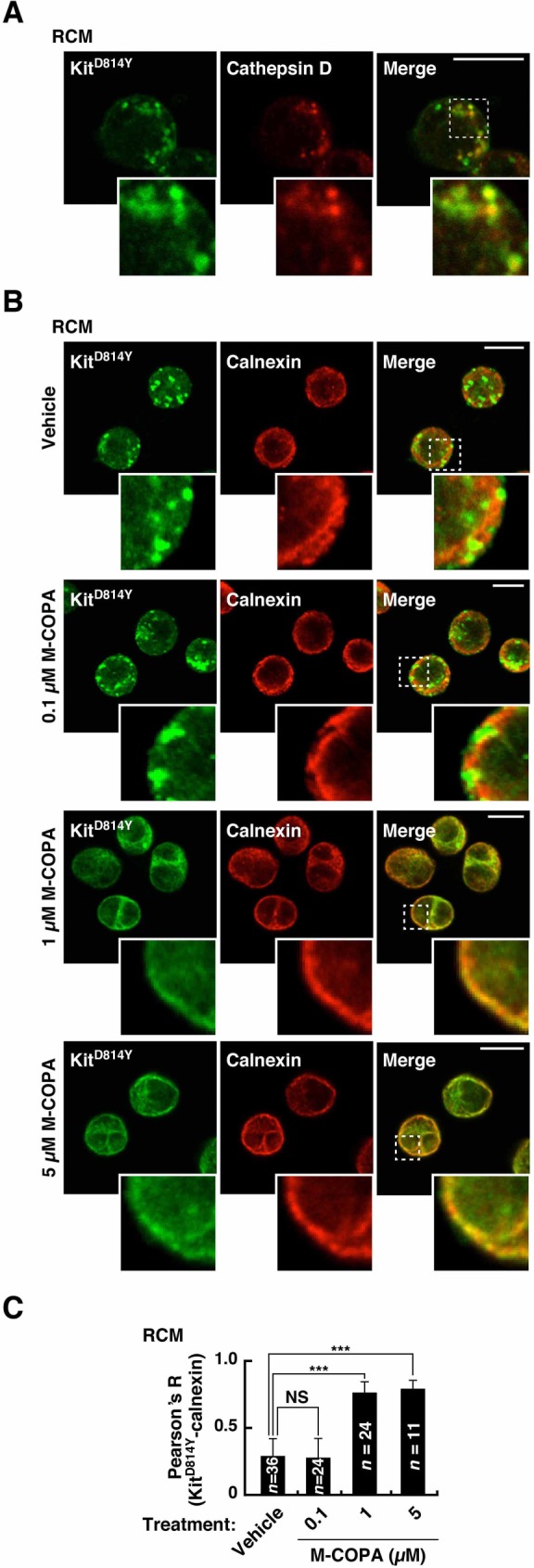
In neoplastic mast cells, M-COPA inhibits Kit trafficking from the ER, resulting in a decrease in endolysosomal Kit. (**A**) RCM cells were immunostained with anti-Kit (green) and anti-cathepsin D (endolysosome marker, red). Insets indicate magnified images of the boxed area. Bar, 10 μm. (**B**) RCM cells were treated with vehicle or 0.1~5 μM M-COPA for 16 hours, then stained for Kit (green) and calnexin (ER marker, red). Insets indicate magnified images of the boxed area. Bars, 10 μm. (**C**) Pearson’s R correlation coefficients were calculated by intensity analysis of Kit *vs*. calnexin. Results are means ± SD (*n* = 11~36). Data were subjected to one-way ANOVA with Dunnett’s multiple comparison *post-hoc* test. ***P < 0.001; NS, not significant. Note that in RCM cells, co-localization of Kit with calnexin was significantly increased by M-COPA treatment.

We previously reported that in neoplastic mast cells, Kit-dependent activation of Akt and STAT5 occurs on endolysosomes and the ER, respectively, and that inhibition of Akt is sufficient for suppression of cell proliferation [29]. Recent studies showed that M-COPA has an anti-cancer effect in Met tyrosine kinase-addicted cancers *in vivo* through inhibition of the trafficking of the receptor to the PM [31–33]. Therefore, we investigated the effect of M-COPA on oncogenic Kit signalling in neoplastic mast cells. Immunofluorescence confocal microscopic analysis showed that in RCM and HMC-1.2, Kit co-localized with the endolysosome marker cathepsin D (Fig 2A and S2A Fig), as previously described [29]. M-COPA decreased the endolysosomal localization of Kit in a dose-dependent manner (Fig 2B and S2B Fig). With Pearson’s R correlation coefficient intensity analysis, we found that M-COPA significantly increased co-localization of Kit with the ER marker calnexin (Fig 2C and S2B Fig), indicating that the trafficking of Kit from the ER is inhibited by M-COPA treatment. Taken together with the fact that Kit^mut^ localized to endolysosomes through endocytosis from the PM after moving along the secretory pathway [29], these results suggest that M-COPA decreases endolysosomal Kit through blocking the biosynthetic transport of Kit from the ER.

### M-COPA can inhibit Akt activation through blocking the localization of Kit^mut^ to endolysosomes in naoplastic mast cells

**Fig 3:**
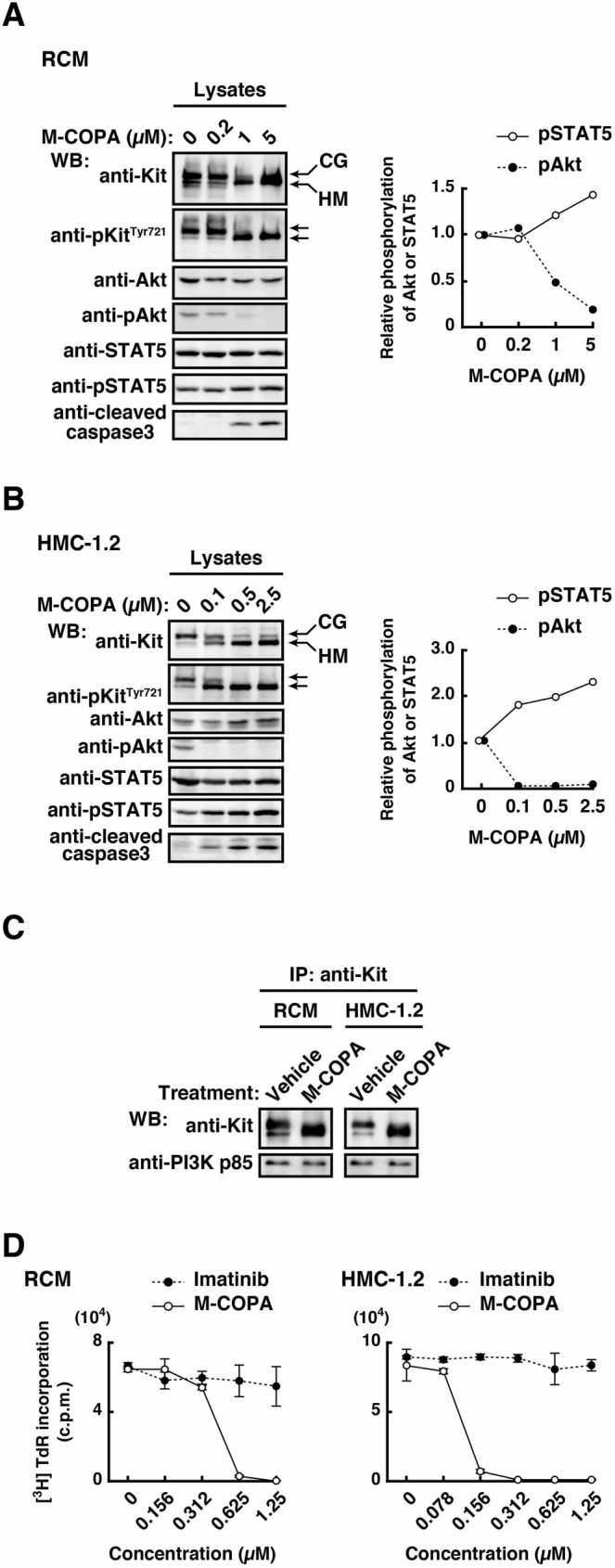
In neoplastic mast cells, M-COPA inhibits Kit-dependent Akt activation through blocking the biosynthetic transport of Kit from the ER. (**A**-**C**) RCM (**A**) and HMC-1.2 (**B**) were treated for 16 hours with vehicle (0) or 0.1~5 μM M-COPA. Cell lysates were immunoblotted with anti-Kit, anti-phospho-Kit^Tyr721^ (anti-pKit^Tyr721^), anti-Akt, anti-pAkt, anti-STAT5, anti-pSTAT5, and anti-cleaved caspase-3. The graphs show the levels of pSTAT5 (open circles) or pAkt (closed circles) expressed relative to lysate from vehicle-treated cells. CG, complex-glycosylated form; HM, high mannose form. (**C**) RCM and HMC-1.2 were treated with 5 μM M-COPA for 16 hours. Anti-Kit immunoprecipitates were immunoblotted with anti-Kit and anti-PI3K p85. Note that M-COPA inhibited Akt activation without affecting the phosphorylation of Kit at Tyr721 or the association of Kit with PI3K p85. (**D**) RCM (left) and HMC-1.2 (right) were treated with vehicle (0), M-COPA (open circles) or imatinib (closed circles) for 24 hours. Proliferation was assessed by [^3^H]-thymidine incorporation. Results (c.p.m.) are means ± SD (*n* = 3).

Next, we tested whether M-COPA suppressed oncogenic activation of Akt through blocking Kit trafficking in RCM and HMC-1.2. As shown in Fig 3A and 3B, Kit shifted to a lower molecular weight form on M-COPA treatment. Since partially glycosylated receptors in the ER subsequently move to the Golgi apparatus for further glycosylation [24,29,36,37], these results support our observation that M-COPA blocks Kit trafficking from the ER. Phosphorylation of Kit at Tyr721, which is critical for activation of the PI3K-Akt pathway [5,10,38], was unaffected by M-COPA treatment (Fig 3A and 3B), indicating that phosphorylation of the tyrosine residue occurs on the ER. Furthermore, using a co-immunoprecipitation assay, we tested whether M-COPA decreased the association of Kit with PI3K. Fig 3C shows that the PI3K p85 subunit was co-immunoprecipitated with Kit, and that ER accumulation of Kit by M-COPA did not affect the association of Kit with p85. Interestingly, M-COPA markedly decreased the activation of Akt, and the effect was correlated with a decreased ER export of Kit (Fig 3A and 3B). These results indicate that in the ER, the Kit-PI3K complex is unable to activate Akt. On the other hand, STAT5 phosphorylation was enhanced by M-COPA treatment (Fig 3A and 3B), supporting our previous finding that Kit^mut^ activates STAT5 on the ER in neoplastic mast cells [29]. Similar results were obtained with RCM cells treated with a well-known ER-to-Golgi trafficking inhibitor brefeldin A (BFA) (S3A–S3C Fig), indicating that through blocking ER export of Kit^mut^, M-COPA inhibits and enhances the activation of Akt and of STAT5, respectively.

Next, we determined whether M-COPA inhibited Akt activation through blockade of Kit glycosylation or Kit trafficking. Previously, we reported that bafilomycin A1 (bafA1) inhibits Kit-dependent Akt activation through endosome-endolysosome trafficking [29] (S4A Fig). Thus, we treated Kit from bafA1-treated cells with endoglycosidase H (endo H), which digests immature glycan but not mature glycan. In bafA1-treated cells, a majority of Kit was not digested by endo H, indicating that Kit^mut^ cannot activate Akt before reaching endolysosomes even if Kit glycosylation is normal (S4B Fig). These results suggest that M-COPA inhibits the Kit-Akt pathway through blocking Kit localization to endolysosomes, probably rather than through inhibiting Kit glycosylation.

### In neoplastic mast cells, M-COPA induces apoptosis through inhibiting Kit-dependent Akt activation that occurs only on endolysosomes

S5A and S5B Fig show that blockade of Akt with Akt inhibitor VIII (Akti VIII) suppressed RCM proliferation and induced apoptosis. Furthermore, induction of apoptosis by M-COPA was correlated with the decrease in Akt activation (Fig 3A and 3B, bottom panels). To confirm whether M-COPA induced apoptosis through inhibiting Akt phosphorylation, we treated a Kit^mut^-negative lung adenocarcinoma cell line A549 with M-COPA. As shown in S5C Fig, blockade of ER export with M-COPA did not result in Akt inhibition and caspase-3 cleavage in A549 cells, supporting our finding that the drug can cause apoptosis through inhibition of the Kit-PI3K-Akt pathway. As shown in Fig 3D, RCM and HMC-1.2 were unable to proliferate in the presence of M-COPA because they underwent apoptosis. These results suggest that M-COPA inhibits the growth of neoplastic mast cells through blocking the anti-apoptotic activation of the Kit-Akt pathway that occurs only on endolysosomes.

### In neoplastic mast cells, Kit^mut^ can activate Erk not only on the ER but also on other compartments

**Fig 4:**
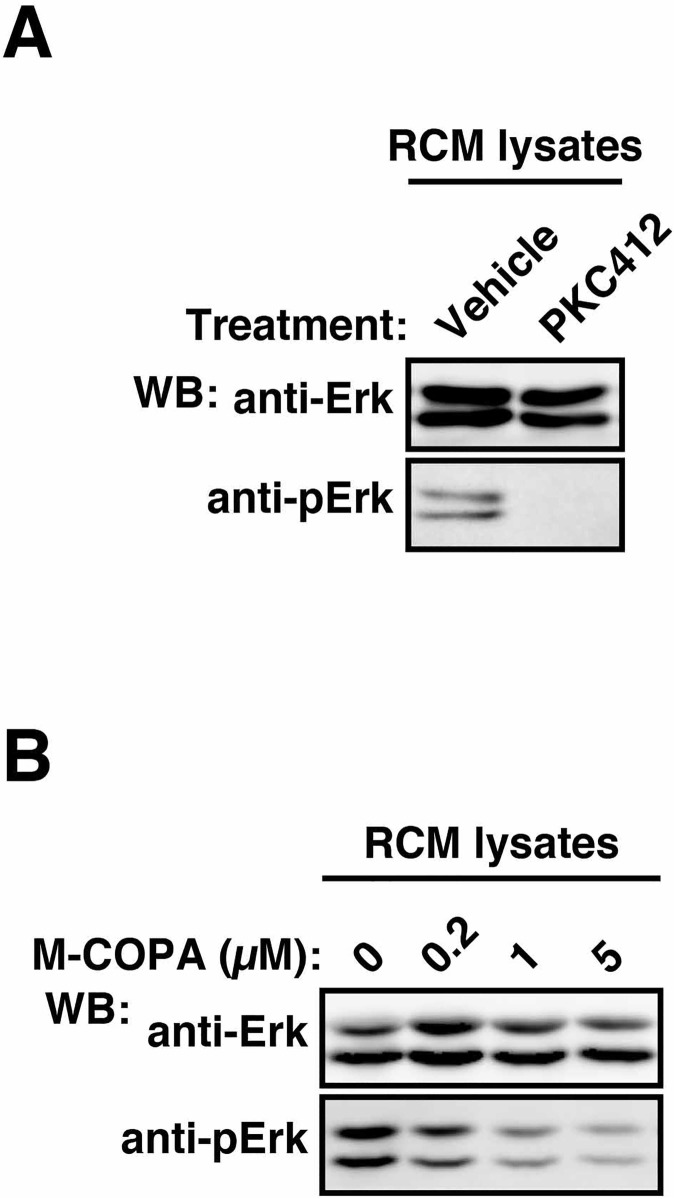
Effect of M-COPA treatment on Kit-dependent Erk activation in RCM cells. (**A** and **B**) RCM cells were treated with vehicle (0), (**A**) 1 μM PKC412 for 24 hours, or (**B**) 0.2~5 μM M-COPA for 16 hours. Cell lysates were immunoblotted with anti-Erk and anti-phospho-Erk (anti-pErk).

Previous studies showed that Kit activates Erk in various cells [4,5,11]. Indeed, Kit^mut^ knockdown with siRNA and Kit inhibition with PKC412 decreased Erk phosphorylation in RCM cells (Fig 4A and S6A Fig), confirming that Kit^mut^ activates Erk in RCM cells. We thus investigated the effect of M-COPA on Kit-dependent Erk activation. In RCM cells, Erk phosphorylation was decreased but remained in M-COPA-treated cells (Fig 4B). Similar results were obtained with BFA-treated RCM cells (S6B Fig). Next, we tested the effect of bafA1 (inhibitor of endosome trafficking) or monensin (inhibitor of Golgi export) on Erk activation. As shown in S6C Fig, similar to M-COPA, these inhibitors had only a partial inhibitory effect on Erk phosphorylation. Although we could not determine the signalling platform for the Kit-Erk pathway in this study, these results indicate that Kit^mut^ can activate Erk not only on the ER but also on other organelles. We previously reported that Erk inhibition by U0126 does not affect proliferation of RCM cells [29]. However, it is possible that the Kit-Erk pathway in neoplastic mast cells plays a role in other cellular events, such as migration and degranulation [39]. Thus, further study will be required for understanding the role of the Kit-Mek-Erk pathway in the pathogenesis of mast cell tumours.

### In neoplastic mast cells, Tyr568/570, Tyr703, Tyr721, and Tyr936 in Kit^mut^ are phosphorylated on the ER

**Fig 5:**
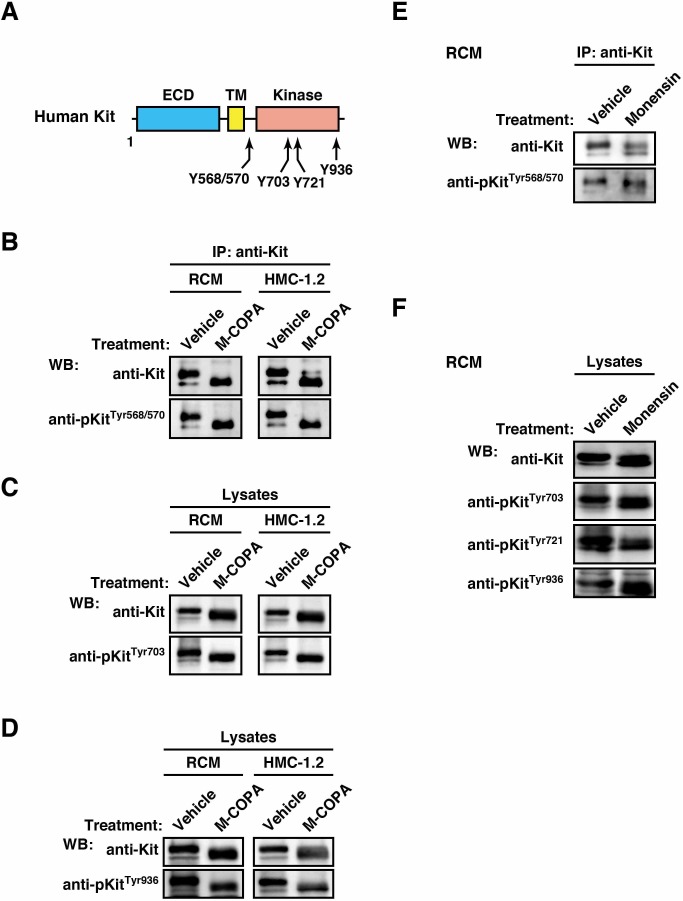
In neoplastic mast cells, Kit phosphorylation at Tyr568/570, Tyr703, Tyr721, and Tyr936 occurs on the ER. (**A**) Tyrosine phosphorylation sites in human Kit. (**B**-**F**) Cells were treated with vehicle or (**B**-**D**) 5 μM M-COPA for 16 hours or (**E** and **F**) 250 nM monensin for 24 hours. Anti-Kit immunoprecipitates (**B** and **E**) and cell lysates (**C**, **D**, and **F**) were immunoblotted with anti-Kit, anti-phospho-Kit^Tyr568/570^ (anti-pKit^Tyr568/570^), anti-pKit^Tyr703^, anti-pKit^Tyr721^, and anti-pKit^Tyr936^. Note that ER-localized Kit was phosphorylated at Tyr568/570, Tyr703, Tyr721 and Tyr936 in neoplastic mast cells.

The critical tyrosine phosphorylation sites in Kit responsible for its downstream activation are in the juxtamembrane region, the kinase domain, and the carboxy-terminal region (referred to henceforth as pTyr568/570, pTyr703, pTyr721, and pTyr936, see Fig 5A) [5–8,10,40]. Previously, we reported that in GIST cell lines, Tyr568/570 and Tyr703 in mutant Kit are dephosphorylated on the ER [24]. Thus we examined whether these five tyrosine residues were phosphorylated on the ER in neoplastic mast cells. Interestingly, pTyr568/570, pTyr703, and pTyr936 remained both in M-COPA-treated and BFA-treated cells, similar to pTyr721 (Fig 5B–5D, and S3D and S3E Fig; see also Fig 3A and 3B). In addition, phosphorylation of these tyrosine residues was unaffected by treatment with monensin, an inhibitor of Golgi export (Fig 5E and 5F). These results suggest that in neoplastic mast cells, the five tyrosine residues in Kit^mut^ are phosphorylated on the ER, not after reaching the PM.

## Discussion

**Fig 6:**
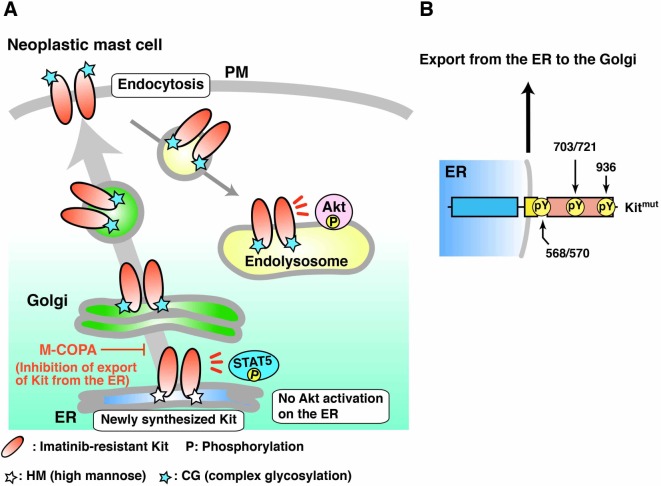
Model of the effect of M-COPA on Kit signalling and on autophosphorylation. (**A**) In neoplastic mast cells, mutant Kit is trafficked to the PM along the secretory pathway and then moves to endolysosomes through endocytosis. Kit activates Akt only on endolysosomes as previously described (Obata et al., 2014). Note that M-COPA inhibits the activation of Akt through blocking ER export of Kit. STAT5 activation is enhanced by M-COPA because it is activated by ER-localized Kit. HM, high mannose; CG, complex glycosylation. (**B**) Tyr568/570, Tyr703, Tyr721, and Tyr936 in Kit are phosphorylated on the ER in neoplastic mast cells.

In this study, we demonstrate that M-COPA inhibits autonomous proliferation of neoplastic mast cells through blocking the trafficking of Kit^mut^ from the ER since oncogenic activation of the Kit-PI3K-Akt pathway occurs only on endolysosomes (Fig 6A). Furthermore, results of our M-COPA treatment assay show that Kit Tyr568/570, Tyr703, Tyr721, and Tyr936 are phosphorylated on the ER, not after reaching the PM (Fig 6B). In addition to previous reports of a leukemia therapy using inhibition of Flt3 trafficking [30,37], our study shows that inhibition of mutant receptor trafficking represents a promising strategy for the treatment of cancers.

Previous studies showed that cancer-causing receptors accumulate and initiate oncogenic signals on intracellular compartments [41,42]. PDGFRα^V561D^ expressed in HEK293 and FGFR3^K650E^ in multiple myeloma cells accumulate on the Golgi apparatus where they activate downstream molecules such as Akt, STATs, and Erk [41,43–45]. EGFR^L858R^ in lung adenocarcinoma and gp130(ΔYY) and Met in hepatoma cause oncogenic signalling on endosomes [42,46–50]. In addition, the localization of receptors bearing secondary mutations that confer a drug resistance is similar to that of receptors bearing primary mutations [24,46,47]. Considering that all mutant receptors are biosynthetically trafficked from the ER and that M-COPA can block the localization of receptors to the signalling platforms, the drug may inhibit their signalling. Our study will help to develop a strategy for the treatment of cancers that have receptors with secondary mutations.

Similar to mast cell tumours, GISTs frequently have constitutively active mutations. Mutant Kit in GISTs is localized and activated on the perinuclear compartment characterized as the Golgi apparatus [24,51,52]. Unlike mast cell tumours, Kit activates Akt, STAT5, and Erk only on the Golgi apparatus in GISTs [24]. Although its sub-cellular localization in GISTs is different from that in mast cell tumours, M-COPA may inhibit oncogenic signalling through blocking the localization of Kit to the signalling platform. Investigation of the effect of M-COPA on GIST growth is now under way.

In neoplastic mast cells, Kit Tyr568/570 and Tyr703 are phosphorylated, whereas in GISTs, the tyrosine residues are dephosphorylated [24]. This may explain why Kit^mut^ in the ER can activate STAT5 and Erk only in mast cell tumours. Since in GIST cells, mast cell tumour-type Kit mutant (Kit^D814Y^) is localized and autophosphorylated on the Golgi apparatus [24], the host environment may determine the Kit signaling platform. Further study will be required for understanding the differences between mast cell tumours and GISTs in the role of ER-localized Kit.

Several inhibitors of protein trafficking, such as brefeldin A (BFA), Exo1, Exo2, and AG1478, have been reported [53–56]. However, BFA has less stability *in vivo* while the others exhibit weaker inhibition of cell growth, and their development as drugs has not progressed. On the other hand, M-COPA had an anti-cancer effect *in vivo* experiments using breast cancer or gastric cancer xenograft models [32,33], and it is now considered a novel anticancer drug candidate. Together with the fact that M-COPA suppressed imatinib-resistant Kit signalling, inhibition of receptor trafficking is a promising approach for the treatment of cancers bearing a resistant mutation to targeting therapy.

## Conclusion

We demonstrate that M-COPA can suppress the proliferation of neoplastic mast cells through blockade of Kit^mut^ trafficking to the signalling platform such as at endolysosomes. Furthermore, this study shows the phosphorylation states of Kit^mut^ on the ER. Our observations will open new fields for developing therapies for cancers that express mutant Kit, such as mast cell tumours, GISTs, acute myeloid leukemia, and melanomas and will be helpful for understanding the spatiotemporal regulation of tyrosine phosphorylation signalling.

## Supporting information

S1 Fig Kit^D814Y^ is essential for autonomous proliferation of RCM cells. (**A** and **B**) RCM cells were transfected with control siRNA or Kit siRNAs (Kit1 or Kit2) and cultured for 20 hours. (**A**) Cell lysates were immunoblotted with anti-Kit and anti-phospho-tyrosine (anti-pTyr). Total protein levels were confirmed by Coomassie staining. (**B**) The graph shows the levels of [^3^H]-thymidine incorporation into RCM cells. Results (c.p.m.) are means ± s.d. (*n* = 3). Data were subjected to one-way ANOVA with Dunnett’s multiple comparison *post-hoc* test. ***P < 0.001.
(EPS)

S2 Fig M-COPA inhibits Kit trafficking from the ER in HMC-1.2 cells. (**A**) HMC-1.2 cells were immunostained with anti-Kit (green) and anti-cathepsin D (endolysosome marker, red) Insets indicate magnified images of the boxed area. Bar, 10 μm. (**B**) HMC-1.2 cells were treated with vehicle or 0.5~5 μM M-COPA for 16 hours, then stained for Kit (green) and calnexin (ER marker, red). Insets indicate magnified images of the boxed area. Bars, 10 μm. The graph shows Pearson’s R correlation coefficients calculated between Kit and calnexin. Results are means ± SD (*n* = 14~30). Data were subjected to one-way ANOVA with Dunnett’s multiple comparison *post-hoc* test. ***P < 0.001. Note that in HMC-1.2 cells, co-localization of Kit with calnexin was significantly increased by M-COPA treatment.
(EPS)

S3 Fig Effect of BFA on Kit trafficking and oncogenic signalling. (**A**) RCM cells were treated with vehicle or 5 μM BFA for 16 hours, then immunostained with anti-Kit (green) and anti-calnexin (ER marker, red). Bars, 10 μm. (**B**-**E**) RCM cells were treated for 16 hours with vehicle (0) or 1~5 μM BFA. (**B**) Cell lysates were immunoblotted with anti-Kit, anti-phospho-Kit^Tyr721^ (anti-pKit^Tyr721^), anti-Akt, anti-pAkt, anti-STAT5, anti-pSTAT5, and anti-cleaved caspase-3. The graph shows the levels of pSTAT5 (open circles) or pAkt (closed circles) expressed relative to lysate from vehicle-treated cells. (**C**-**E**) RCM cells were treated with 5 μM BFA for 16 hours. Anti-Kit immunoprecipitates (**C** and **D**) or lysates (**E**) were immunoblotted with the indicated antibody.
(EPS)

S4 Fig Blockade of Kit trafficking to endolysosomes inhibits Akt activation. (**A** and **B**) RCM cells were treated with vehicle or 100 nM BafA1 for 24 hours. (**A**) Lysates were immunoblotted with the indicated antibody. (**B**) Lysates were treated with peptide N-glycosidase F (PNGase F) or endoglycosidase H (endo H) then immunoblotted. CG, complex-glycosylated form; HM, high mannose form; DG, deglycosylated form.
(EPS)

S5 Fig Inhibition of Akt induces apoptosis in RCM cells. (**A**) RCM cells were treated with vehicle (0), or Akt inhibitor VIII (Akti VIII) for 24 hours. Proliferation was assessed by [^3^H]-thymidine incorporation. Results (c.p.m.) are means ± SD (*n* = 3). (**B**) Immunoblots, lysates from RCM cells treated with vehicle or 10 μM Akti VIII for 24 hours. Note that Akt inhibition induced apoptosis in RCM cells. (**C**) A549 or HMC-1.2 were treated with vehicle (0) or 1~5 μM M-COPA for 16 hours. Lysates were immunoblotted. Total protein levels were confirmed by Coomassie staining. Note that M-COPA did not affect the Akt activation and cleavage of caspase-3.
(EPS)

S6 Fig Effect of inhibition of Kit trafficking on Erk activation. (**A**) RCM cells were transfected with control siRNA or Kit siRNAs (Kit1 or Kit2) and cultured for 20 hours. Cell lysates were immunoblotted with anti-Erk and anti-phospho-Erk (anti-pErk). (**B** and **C**) RCM cells were treated with (**B**) vehicle (0), 1~5 μM BFA for 16 hours, (**C**) 250 nM monensin or 100 nM BafA1 for 24 hours. Cell lysates were immunoblotted.
(EPS)
